# Book Review: Teacher Wellbeing

**DOI:** 10.3389/fpsyg.2021.707765

**Published:** 2021-06-30

**Authors:** Jian Fan

**Affiliations:** Center for Second Language Writing Research, Henan University, Kaifeng, China

**Keywords:** positive teacher, teacher well-being, accomplishment, habits of thinking, positive psychology

Rooted in positive psychology, positive teacher well-being has recently gained enormous momentum in teacher education. Research has also substantiated that although language teaching is rewarding, it is also anxiety-inducing, and teachers need to bear many negative emotions and pressures in their professional careers. Besides, it is postulated that teacher well-being is inextricably linked to students' outcomes, achievement, motivation, and engagement. Consequently, it is immensely essential to give full attention to language teacher well-being. Nonetheless, research in this area remains scanty. The present monograph, entitled *Teacher Wellbeing*, eloquently written by Sarah Mercer and Tammy Gregersen, is an opportune addition to the literature on teacher education since it views teachers' professional development and well-being through the prism of strengths-driven, opportunity-focused, and internally driven processes.

Structurally, this trailblazing monograph encompasses eight chapters that are thematically interconnected. In Chapter 1, conceptualizing the concept of well-being, the authors elucidate that some frameworks shed light on our understanding of well-being throughout the book. The first framework, known as PERMA, which stands for Positive emotion, Engagement, Relationships, Meaning, and Accomplishment was conceptualized by Seligman (2011). The second underlying rationale behind various approaches of well-being in this volume is the eudemonic view of well-being, which accentuates a sense of meaning, purpose, fulfillment of one's potential, and connection in the real world. The third underlying framework fleshes out that individuals have four different types of well-being: physical, emotional, mental or intellectual, and spiritual.

Pinpointing the indispensable role of institutions and what they can offer to boost the well-being of their staff, facilitate job satisfaction, as well as diminish the amount of burnout is the focus of Chapter 2. The authors highlight that institutions need to appreciate the benefits for their organization, their staff, and their students, to foster teacher well-being. The chapter presents different characteristics of positive organizations which render them rewarding places to work, discusses how job fulfillment can be enormously impacted by the “person–organization fit,” and stresses the significance of teacher autonomy in their work-related practices.

Chapter 3 concentrates on habits of thinking and how to avoid counter-productive thoughts to give rise to conducive support. The authors also discuss diverse identities involved in being a language teacher, introduce the notion of mindsets, as ways of thinking about personality traits, and demonstrate the reasons individuals give for their successes. Considering the sources of motivation for language teachers and how teachers can maintain and protect motivation in a professional career is the prime objective of Chapter 4. The authors elaborate on how flow motivation boosts their spirit momentarily and builds psychological capital over time. The authors cogently argue that dips in motivation are prevalent, so finding moments of flow, job crafting, and reflecting on personal strengths, can provide some remedies to nurture our well-being as a language teacher.

One of the cornerstones of teacher well-being is to nurture and nourish positive social relationships in the workplace. Chapter 5 scrutinizes the quintessential elements which lead to positive relationships, emerging primarily from socio-emotional competencies. This chapter is particularly fascinating because it gives more information about how to build and broaden rapport with learners, from the interpersonal and intercultural dimensions of relationships. The chapter also investigates ways of enhancing positive group dynamics and the value of collaborative activities.

Foregrounding the pivotal role of experiencing and realizing a wide array of emotional dimensions in language teaching is the focus of Chapter 6. The authors aptly caution against an undue emphasis on negative emotions and suggest how to embark on positive dimensions. What I found particularly intriguing in this chapter was the concept of emotional labor, defined as a kind of emotion regulation where an individual terminates an emotion and performs another emotion. The penultimate chapter not only deals with the concept of stress but also examines what an optimal level of stress can be for each individual, regarding different personalities, life commitments, and cultural time perceptions. The chapter also critically discusses the concept of work-life balance, pinpointing the importance of physical well-being for mental well-being. It discusses the lessons to be learned about how to consciously manage our mental and physical well-being through practices of mindfulness.

In the closing chapter, the authors look ahead at how to nurture a sense of balance in well-being in the long run. Short-term interventions and setting goals can help us keep abreast of our manifold range of individual priorities without losing sight of our overall well-being. Discussing what language teachers can do to nurture their well-being in a long-lasting way for the rest of their professional careers and its benefits of being a language teacher is the other objective of this chapter. The authors provide a set of “questions for action” that reiterate crucial themes of all chapters.

The merits of this unique volume are manifold. First, it offers language teachers viable support in nurturing their well-being by highlighting the value of our emotional, physical, psychological, social, personal, and professional selves through a “whole-person” perspective regarding contextual factors. Secondly, judiciously embedded throughout the volume are some inspirational quotes that capture the essence of fundamental points about well-being. Thirdly, what seems applicable is the activities at the end of each chapter which can help language teachers to reflect on their personal growth and well-being. Finally, recommending three books at the end of each chapter provides a treasure trove of information for those enthusiasts to upgrade and deepen their understanding of the pertinent literature on well-being. However, had the authors added more empirical first-hand research on well-being, the readers would have benefited enormously.

All in all, I feel confident to suggest this thought-provocative volume to in-service and pre-service teachers, school principals, teacher educators, policymakers, and syllabus designers who are interested in nurturing and sustaining language teacher well-being. I suppose more studies can be done on the role of well-being and other factors such as resilience, immunity, job satisfaction, etc.

## Author Contributions

JF contributed to conception and design of the study, wrote the first draft of the manuscript, then wrote sections of the manuscript and approved the submitted version.

## Conflict of Interest

The author declares that the research was conducted in the absence of any commercial or financial relationships that could be construed as a potential conflict of interest.
